# The Bradshaw Lecture on Subjective Sensation of Sound

**Published:** 1896-11-14

**Authors:** W. R. Gowers

**Affiliations:** Consulting Physician to University College Hospital; Physician to the National Hospital for the Paralysed and Epileptic.


### THE BRADSHAW LECTURE ON SUBJECTIVE SENSATION OF SOUND.

Delivered before the Royal College of Physicians of London on November 5th, 1896, by W. R. Gowers, M.D.Lond., F.R.S., Consulting Physician to University College Hospital; Physician to the National Hospital for the Paralysed and Epileptic.

[Abstract.]

After referring in feeling terms to the late President of the College, whom he spoke of as his oldest medical friend, Dr. Gowers said he proposed to consider some features of the sounds that were heard when no external cause for them existed—subjective auditory sensations—and to present some of the more salient facts that had come under his notice regarding the two chief forms of these sensations, those of labyrinthine and of central origin. No orderly arrangement was possible. The facts themselves not only were so various and varied as to baffle adequate classification, but they presented so tangled a skein, or rather network, that their separation was not possible except by force. To tear asunder that which was interwoven was an easy means of distinction. But we must be content with a less effective way, and try to trace where we could not disentangle, and to perceive where the interlacing strands were chiefly gathered together. Among the facts that he would have to mention were some relating to the power of hearing certain musical notes. The resemblance which existed between the subjective sensations of vision and those of sound was superficial; the difference was profound and also instructive. Subjective visual sensations were chiefly of central origin, and in themselves chiefly of physiological interest, while those of hearing were to a large extent produced in the sensory organ itself. They were often so obtrusive, and even distressing, that by the sinister dignity of a special name, the symptom had almost come to be looked upon as a disease. The frequency with which the ear, and the rarity with which the eye, was the cause of subjective sensations was also of indirect significance. We were apt to place these two senses much too near together in our thought. Both were, indeed, excited by energy in the form of motion and of wave motion. Hence they presented points of resemblance which might attract more attention than did the difference, and, indeed, distance, between them. Consider—for the question was of importance for us—consider the difference involved in the frequency of the vibrations that act on each organ. If we took a colour about the middle of the visible spectrum and a musical note below the middle of the treble clef, we should find that, in the interval of time between two successive waves of sound, there occurred about one million million waves of light. It was not surprising, therefore, to find that, whereas the waves of sound occurred only in media so definitely material that they could be weighed, the waves of light occurred in a medium which was not only imponderable, but in which that quality was carried up to the hypothetical. Further, the waves of light could be traced upwards until they vanished to our direct perception, though beyond them, and their actinic successors, there seemed to be almost ultra-ethereal waves of such a nature that they could be only designated by the algebraic symbol for that which is unknown; on the other hand, the waves of sound passed down to the coarser motion which could be only felt. It was a familiar fact that all the lower waves of sound could be felt by the sense of touch if only they occurred in a medium sufficiently inelastic. With this was associated yet another difference, the waves of light, like the common waves of water, were transverse to the line of propagation ("transversal" waves, according to the double adjectival form which physicists have devised). But the waves of sound were "direct"; they occurred in the line of propagation, as the motion between billiard balls when one at the end of a row was struck and that at the other end flew off. These "direct" or "end-on" variations (as they might be called) manifestly possessed a greater impetus, and such wave motion was nearer to the simple motion of a mass, by which the nerves of touch were commonly affected. These facts, which pointed to an essential identity between the stimulus of touch and that of sound, were not disturbed in their significance by the special character of many auditory sensations, such as musical notes. Their significance was, moreover, carried further by the facts of development, which showed a common (epiblastic) origin for the structures that subserved each form of sensation; by the facts of anatomy, which showed that sound was received by minute hairs that acted upon the nerves; and by the facts of comparative anatomy, which showed the early form of the ear to consist simply of hairs upon the skin connected with a special nerve, homologous with that of hearing in higher animals. This relation of hearing to the sense of touch removed one apparent anomaly—the fact that one part of the auditory nerve seemed to have nothing to do with hearing. Although we could not say that sound did not act upon the structures of the semi-circular canals, we could not doubt that their chief function was to cause nerve impulses to be produced by the simple motion of the fluid within them and its varying pressure on the hairs in their dilated ends. We had in this a response to the very simplest form of motion, quite as simple as that which excited the nerves of touch. The impulses produced in the semi-circular canals did not act upon consciousness, although their effects might do so. This should teach us not to regard conscious sensation as a test of the nerve impulses that might be produced in other structures of the ear.

His hearers would know that tinnitus might be a continuous sound or pulsating, and that in the latter case the pulsations often corresponded to the arterial waves. They would know also how many and extensive were the variations in character, from the simplest to the most elaborate sound. The very frequent coincidence of these sounds with evidence of ear disease, varying in seat and character, but usually involving the labyrinth and commonly limited to it, compelled the conclusion that the most common source of these sounds was the internal ear, in which the auditory nerve was normally excited. But we had cases, especially those in which the sensations were the warning of epileptic fits, in which we must regard the auditory centre in the cortex of the brain as their source, and in rare cases the correctness of this conclusion had been proved by the discovery of organic disease in this region. The question arose, Why was the subjective sound referred to the ear and not to the external world? Subjective visual sensations always seemed of outside origin, why did not those of hearing? The answer was complex, and brought before us many points connected with the subject. The apparent source of the sound seemed to be, in part, a matter of mental inference. Continuous sensations—hissing or buzzing, for instance—seemed as a rule to be of external origin when they were first observed, but their persistence soon convinced the sufferer that they could not be; knowing that they must have their origin in the ear, he ceased to refer them to an external cause, and they then seemed to him to be felt in the ear, although often, if he could separate the sensation from the inference, he could still for a moment realise that they were due to something outside. It was so also with brief, unfamiliar sounds. At first they seemed to have an external cause, but their repetition involved the knowledge that they had not, and the knowledge so acted on consciousness that their aural source seemed to be felt. An instance of this was afforded by a patient who was subject occasionally to the sound of a bell several times repeated. When he first became liable to the sound he actually sent a message to his next-door neighbour asking that the clock might be stopped, the sound of which so much annoyed him, and he only discovered the subjective nature of the sound when he was informed that the neighbouring house did not contain a single striking clock. An instance of persistent reference to an external cause was a girl subject to a sound so perfectly resembling two or three taps at a door, that, in spite of her past experience of this subjective nature, she frequently called out, "Come in." Another was a woman who had long been liable to attacks of a rumbling sound, each lasting from a quarter to half a minute, but was never able to distinguish them from distant thunder. Even after she had long known of their subjective nature she would ask persons near her whether they did not hear distant thunder. More elaborate sounds generally seemed external, but, on the other hand, pulsating tinnitus was almost always referred to the ear even from its commencement.

The point next to be considered was the reference of subjective sounds to the head and not to the ear. Sensations produced in one labyrinth were sometimes referred only to some one part of the head on that side. In one case of left-sided labyrinthine deafness, the sound was always referred to the left side of the head, but it seemed to spread when loud through the whole head; it was never referred to the ear. In other cases he had met with, it had been referred to the parietal region, to the parietal and occipital, to the occipital only, and in one with considerable nerve deafness a persistent rumbling sound was always referred to the region between the temple and the vertex, never to the ear or any other part. In these cases of one-sided head-sound the extension to the whole head, when the sound became louder, was very common.

It was a curious fact that the subjective sounds which originated in the auditory centre in the cortex were referred to the same seats as those of labyrinthine origin. This was frequently the case with the central sounds—those that occurred as the warning of epileptic fits. When these were elaborate they were referred to the external world, but when they were simple or "crude," they generally seemed to the patient to be produced either in the head or in one or both ears. As examples he might mention a whistling sound referred to both ears, a similar sound referred to both sides of the head above the ears, a buzzing sound which seemed to pass through the head from one ear to the other, a whistle referred to the ear on the side on which the subsequent unilateral convulsion occurred, and a whistle seemingly at the top of the head in one case and in another at the occiput. One feature, which promised occasionally to be of definite practical importance, was the relation of the tinnitus to external sounds. In the majority of cases the subjective sound was heard most when there was silence, sometimes it was noticed only then. There were cases, however, in which external sounds increased the tinnitus, and some of these were both curious and noteworthy. There might be a peculiar hyperacusis, by which certain sounds seemed especially loud and unpleasant, and seemed to increase the subjective sound. A question of some practical importance was the induction of tinnitus by sound. It was not often well marked, especially in such tinnitus as could be reasonably regarded as labyrinthine, but such cases occurred, and an important point was the beneficial influence of silence upon them. A clergyman aged fifty-four years, certainly gouty, began to suffer, at the age of fifty, from gradual deafness of the right ear, tinnitus, and attacks of vertigo. An aural surgeon found the meatus and middle ear to be normal. The deafness was almost complete to high notes, $C^2$ being heard but not $C^3$, the voice being, however, heard fairly well. The subjective sound was continuous in character, but it was slight or absent when there was external silence, and was excited by any loud sound of more than brief duration. A short ride in a cab would make it loud, although at starting he had been free. The effect of the music in his church was so great that for a time he had to abstain from duty, and the influence of his own voice, if he read aloud for an hour, was distressing. Either influence would cause the sound to become such that he compared it to "the blowing off of a full head of steam in a locomotive." Even then, if he retired to his room, in perfect silence, the sound would gradually lessen, and in the course of two hours would cease entirely. In this case great benefit was obtained by following the indication thus afforded and securing prolonged freedom from external sound. A large number of the cases in which there was aural tinnitus were also affected with vertigo, and this was often associated with very curious varieties of noises in the ears.

It was strange how few facts we had regarding the pathological changes which underlay the symptoms in the common class of cases, where there was some deafness, tinnitus, and often vertigo, gradual in onset, and often slowly progressive. It was strange, because our Poor-law infirmaries and workhouses contained always many cases of the kind, because modern methods had increased so much the revealing power of the microscope, and because the present generation of workers were not prone to leave neglected any unexplored field. But we could surmise much with reasonable sureness. In cases with striking and unusual symptoms, conspicuous changes had been found, concretions and the like. Further, we knew that the parts chiefly concerned were of a fibrous or fibro-epitheliated structure, in which epithelial hairs received the sound vibrations, and in some way these excited nerve impulses in the delicate structures by which the two were united, and we knew how minute a structural alteration might suffice to derange this mechanism. We knew, also, that this membranous labyrinth might be the seat of acute inflammations, of which the most significant was that which occurred as the effect of a catarrhal blood state, one of those peculiar toxic states which had a special local incidence, for the inflammation was limited and symmetrical. It was now generally recognised that when there was complete deafness as the sole result of what was regarded as an attack of meningitis in early life, the malady was not meningitis but bilateral inflammation ot the labyrinth, the general cerebral symptoms of which closely resemble those of meningitis. We had in this fact, not only the indication of a special blood state, but also of special susceptibility on the part of the labyrinth. We knew from many other facts that such susceptibility to acute inflammations of this kind on the part of fibrous structures involved also a proneness to suffer from chronic changes in later life, especially in cases where there was an inherited rheumatic or gouty tendency, the tendency which might be called "tissue gout." We could thus understand the fact that these chronic labyrinthine changes were especially common in such subjects. We also knew that in late life, apart from such predisposition, the tendency to analogous affections of other fibrous structures was common, as in the great tendency to chronic rheumatism in the old, which we might ascribe to the influence on the tissues of waning vitality, coupled with the effect on the blood of the age-imperfection of the organs on which the blood state chiefly depended. The treatment of these affections constituted one of the most difficult and most obscure branches of therapeutics. This was not, indeed, surprising. Of all slow processes of disease it might be said that the morbid product was the sign of an accomplished fact. Damage and cicatrisation went hand in hand, but the process of cicatrisation was not recovery, and its effects necessarily endured. But we still had some power, and we should be very slow to give up hope or to relax our efforts to obtain a cure. How impressive was the lesson we had just had from science that the word "impossible" should find no place in our vocabulary. Even two years ago, had the wisest among us been asked the question, Is it possible that before the end of the century we should be able to see through a deal door—what would have been the answer? The humility which this should teach should equally prevent us from limiting the possibilities of power to any department of science or to any branch of our art that is founded thereupon.

